# Seasonal variation in blood pressure: what is still missing?

**DOI:** 10.3389/fcvm.2023.1233325

**Published:** 2023-08-11

**Authors:** Eduardo Costa Duarte Barbosa, Giovani Schulte Farina, Carolina Souza Basso, Miguel Camafort, Antonio Coca, Wilson Nadruz

**Affiliations:** ^1^ Hypertension League of Porto Alegre, Porto Alegre, Brazil; ^2^ Department of Internal Medicine, School of Medical Sciences, State University of Campinas, Campinas, Brazil; ^3^ Department of Hypertension and Cardiometabolism, São Francisco Hospital, Santa Casa de Misericórdia de Porto Alegre, Feevale University, Porto Alegre, Brazil; ^4^ Center for Clinical Research and Management Education, Division of Health Care Sciences, Dresden International University, Dresden, Germany; ^5^ School of Medicine, Lutheran University of Brazil, Canoas, Brazil; ^6^ Hypertension and Vascular Risk Unit, Hospital Clínic (IDIBAPS), Department of Internal Medicine, University of Barcelona, Barcelona, Spain; ^7^ Centro de Investigación Biomédica en Red-Fisiopatología de la Obesidad y Nutrición (CIBEROBN), Instituto de Salud Carlos III, Madrid, Spain

**Keywords:** blood pressure, seasonal variation, masked hypertension, white coat hypertension, air pollution, altitude, environment

## Abstract

Seasonal variation of blood pressure (BP) is a topic in cardiology that has gained more attention throughout the years. Although it is extensively documented that BP increases in seasons coupled with lower temperatures, there are still many gaps in this knowledge field that need to be explored. Notably, seasonal variation of BP phenotypes, such as masked and white coat hypertension, and the impact of air pollution, latitude, and altitude on seasonal variation of BP are still poorly described in the literature, and the levels of the existing evidence are low. Therefore, further investigations on these topics are needed to provide robust evidence that can be used in clinical practice.

## 1. Introduction

Seasonal variation of blood pressure (BP) is an important topic in cardiology that has gained more relevance throughout the years. In the treatise titled “*On Airs, Waters, and Places*” (1) Hippocrates had already suggested in 400 BC that to investigate medicine properly, physicians should “*in the first place to consider the seasons of the year, and what effects each of them produces*”. Rose (2) reported in 1961 the first analysis evidencing the influence of BP fluctuations within the seasons. Since then, several epidemiological studies have shown that cold air stimulation can promote an increase in BP (3–5). Additionally, clinical trials indicated an association between seasons with lower temperature and greater cardiovascular mortality (6, 7). In 2020, the ESH Working Group on Blood Pressure Monitoring and Cardiovascular Variability published a consensus statement on seasonal variation of BP (8) summarizing the main evidence on this topic so far. They described several interesting aspects of the pathophysiological mechanisms involving temperature and BP, different strategies for measuring the seasonal variation of BP, the prognostic relevance of seasonal BP changes, clinical implications of this association, and final recommendations for clinical practice. However, we would like to raise some other important gaps that still need further investigation and may contribute to a better understanding of seasonal BP variation. In this review, we will discuss the existing knowledge and the gaps about the association of seasonal variation of BP with different hypertension phenotypes, air pollution, latitude, and altitude.

## 2. Masked hypertension and white-coat hypertension

Hypertension is a major risk factor for cardiovascular events (9, 10) and is usually diagnosed based on office BP measurements. However, due to BP variability, current guidelines have recommended the performance of out-of-office BP measurements besides office BP measurements for a more accurate diagnosis of hypertension (11). The evaluation of office and out-of-office BP in the same subject has revealed that different hypertension phenotypes may exist (11). Sustained hypertension (SH) is usually the most common hypertension phenotype and is coupled with elevated cardiovascular risk (11). Masked hypertension (MH) and white-coat hypertension (WCH) must be also considered, given their high frequency in the population and clinical relevance. MH and WCH may have heterogeneous prevalence in diverse clinical settings and populations, reaching 7%–52% and 9%–54% respectively (12, 13). In this regard, a recent study by Barroso et al. (14) evidenced an expressive rate of misdiagnosis when solely regarding on office BP levels. The authors identified that 20.6% of the participants with an office prehypertension diagnosis had MH, while 27.8% of individuals with office stage-1 hypertension had WCH. Importantly, both MH and WCH are also associated with higher cardiovascular morbidity and mortality when compared with normotension (15).

Because both office BP and out-of-office BP increase in colder seasons, the prevalence of SH is markedly greater in the winter when compared to the summer (8, 16, 17). Conversely, little is known regarding the impact of the seasons on MH and WCH. A *post-hoc* analysis of the Japan Morning Surge Home Blood Pressure (J-HOP) Study (16) evaluating 4,267 individuals found that MH prevalence was lower in the summer compared to the other seasons [MH odds ratio (OR) winter/summer = 2.36 [95% Confidence Interval (CI) = 1.79–3.10], *p* < 0.001] while WCH prevalence was higher in the summer, with an OR winter/summer = 0.55 (95% CI = 0.42–0.72; *p* < 0.001). Likewise, an ambulatory BP monitoring study (18) including 1,075 Japanese patients with chronic kidney disease evidenced that MH was more common in the winter, while WCH was more prevalent in the summer. By contrast, a Chinese study evaluating 649 adolescents assessed by ambulatory BP monitoring reported that both WCH and MH were more frequently detected in the summer (17). Furthermore, Narita et al. (19) found a higher prevalence of nocturnal masked hypertension in the summer among 2,544 Japanese individuals assessed by home BP monitoring. The discrepancies regarding the seasonal variation of hypertension phenotypes reported by the aforementioned studies underscore the need of further studies addressing this topic. In addition, available studies were conducted in Eastern countries and it is not known whether their results may be reproducible in other populations.

## 3. Air pollution

Another important topic is the impact of air pollution on the seasonal variation of BP (20). Both in- and outdoor air pollution are harmful to the cardiovascular system and may increase the BP (20, 21). However, most of the studies on this topic have focused on the short-term effects of air pollution on BP (22, 23). Air pollutants concentration may vary seasonally, depending on the region (24). Thus, some studies reported seasonal differences in the association of air pollution with all-cause and cardiovascular mortality (25–28). Recently, Jin et al. (29) found that environmental ozone was associated with an increased risk of cardiovascular diseases (hazard ratio = 1.0035; 95% CI = 1.0033–1.0037) during the warm months in older Americans.

Air pollutants have been also associated with hospital admissions for hypertension, depending on the climate conditions. Tsai et al. (30) and Chen and Yang (31) found that different air pollutant types were associated with hospitalizations on warm and cool days. Wu et al. (32) conducted a cohort study with young adults and found significant interactions between temperature and air pollution on BP, especially for high air pollution concentrations. The average systolic BP (SBP) change related to a 10°C decrease in temperature for high concentrations of particulate matter with a diameter ≤2.5 μm was 3.6 mmHg (95% CI = 1.9–5.2 mmHg). Similar results were found for organic carbon and nitrogen dioxide, being the average SBP change of 3.3 mmHg (95% CI = 2.0–4.6 mmHg) and 2.8 mmHg (95% CI = 1.6–4.1 mmHg), respectively. A cross-sectional study by Choi et al. (33) also found that summer and winter seasons present correlations of different air pollutants with increased SBP and diastolic BP (DBP) in each season. Fine particulate matter and nitrogen dioxide were associated with BP increase in the warm-weather seasons, while sulfur dioxide and ozone were associated with BP increase in the cold-weather seasons. It is important to note that most of the aforementioned studies were restricted to Easter Asia, which could also limit the external validity of results for other geographical areas with different ethnicities and cultural characteristics. Therefore, further studies about the role and impact of air pollution on BP and its seasonal variability are needed (34).

## 4. Latitude and altitude

BP may vary differently from geographical areas because of differences in latitude and altitude, given that Earth is a geoid. Few studies evaluated differences in seasonal variation of BP according to latitudes, and most of them assessed the effects of temperature and ultraviolet light (35, 36), as well summarized by Weller et al. (37). Of note, Duranton et al. (35) conducted a study in patients undergoing haemodialysis in different latitudes in Europe and found that individuals on northern latitudes had an attenuated seasonal variation of the BP. Interestingly, the same group (38) found no interaction of different latitudes in the seasonal variation of BP when analysing a different cohort of haemodialysis patients, suggesting that knowledge on this topic is far from be established. It is also noteworthy that a substantial part of the evidence evaluating the impact of latitude on seasonal variation of BP was derived from a specific group of patients (hemodialysis patients) and was conducted in Europe. Furthermore, there is a lack of evidence regarding the effects of geomagnetic activity and gravity on the different latitudes and how they could affect BP in the different seasons.

Altitude is also known to interfere with BP. Tsao et al. (39) conducted a cohort study in Taiwan with a small sample of subjects comparing BP in different altitudes in winter and summer. SBP showed a significant variation between altitudes in winter (120.4 ± 17.6 mmHg at 298 m vs. 136.1 ± 19.3 mmHg at 2,610 m; *p* < 0.0001), but not in summer (120.7 ± 13.2 mmHg at 298 m vs. 123.6 ± 17.0 mmHg at 2,610 m *p* = 0.0786). However, DBP variation was significant in both seasons (78.1 ± 11.6 mmHg at 298 m vs. 82.6 ± 10.9 mmHg at 2,610 m *p* = 0.0096, and 78.9 ± 8.9 mmHg at 298 m vs. 76.2 ± 8.9 mmHg at 2,610 m *p* = 0.0022, respectively). To our knowledge, this is the only study we found assessing the effects of altitude on the seasonal variation of BP. Therefore, further studies are necessary to establish the impact of altitude on seasonal BP variation.

## 5. Conclusion

Further investigations are still needed to establish the real impact of seasonal variation of BP on hypertension phenotypes and the role of air pollution, latitude, and altitude on this regard. For all topics, there is an urge for studies with more robust designs—preferably cohorts, using primary datasets (not *post-hoc* analyses), with larger sample sizes, and evaluating broader populations with different ethnicities and cultural characteristics. Hypertension and BP involve the individual’s intrinsic and extrinsic characteristics. Genetic propensity combined with behavioral and environmental factors may result in a predisposition to developing hypertension (40). For that reason, it is important to assess different ethnicities (genetic pools), cultures (dietary intake and other behavioral factors), and geographical areas in studies evaluating BP, especially seasonal variations of BP.

As Hippocrates has written in “*On Airs, Waters, and Places*” (1), it is crucial to understand the environmental factors underlying and associated with diseases. In the case of BP and hypertension, the environment plays a very important role, and, once physicians understand better these aspects, patients will be benefited from more individualized treatments and management.
